# A disintegrin derivative as a case study for PHIP labeling of disulfide bridged biomolecules

**DOI:** 10.1038/s41598-022-06327-z

**Published:** 2022-02-11

**Authors:** Max Fleckenstein, Kevin Herr, Franziska Theiß, Stephan Knecht, Laura Wienands, Martin Brodrecht, Michael Reggelin, Gerd Buntkowsky

**Affiliations:** 1 Institute of Organic Chemistry, Technical University Darmstadt, Alarich-Weiss-Straße 4, 64287 Darmstadt, Germany; 2 Institute of Physical Chemistry, Technical University Darmstadt, Alarich-Weiss-Straße 8, 64287 Darmstadt, Germany

**Keywords:** Biophysical chemistry, Chemical physics, Peptides

## Abstract

A specific labeling strategy for bioactive molecules is presented for eptifibatide (integrilin) an antiplatelet aggregation inhibitor, which derives from the disintegrin protein barbourin in the venom of certain rattlesnakes. By specifically labeling the disulfide bridge this molecule becomes accessible for the nuclear spin hyperpolarization method of parahydrogen induced polarization (PHIP). The PHIP-label was synthesized and inserted into the disulfide bridge of eptifibatide via reduction of the peptide and insertion by a double Michael addition under physiological conditions. This procedure is universally applicable for disulfide-containing biomolecules and preserves their tertiary structure with a minimum of change. HPLC and MS spectra prove the successful insertion of the label. ^1^H-PHIP-NMR experiments yield a factor of over 1000 as lower limit for the enhancement factor. These results demonstrate the high potential of the labeling strategy for the introduction of site selective PHIP-labels into biomolecules’ disulfide bonds.

## Introduction

Besides analytical methods such as circular dichromatic spectroscopy, X-ray crystallography (XRD) or small-angle neutron scattering (SANS) nuclear magnetic resonance (NMR) spectroscopy is the method of choice for most scientists, when structural questions on biomolecules, signaling pathways or biological networks arise^1,2^. Unfortunately, the inherent limitation of NMR analysis is the low sensitivity and a distinct signal broadening in case of macromolecular systems^3^. Therefore, hyperpolarization techniques such as spin exchange optical pumping (SEOP)^4^, dynamic nuclear polarization (DNP)^5^, (parahydrogen induced polarization (PHIP)^6,7^ or SABRE (signal amplification by reversible exchange)^8,9^ gain increased interest in overcoming the low sensitivity of NMR. These methods differ in the source of polarization, which provides the signal enhancement on the investigated nuclei^10–13^. In case of biological structure analysis PHIP-measurements seem to be the most promising method, due to its inexpensive technical set up and easy measurement conditions^14^. Besides a multiple bond, on which a hydrogenation catalyst can hydrogenate, PHIP does not impose any special conditions on the substrate. There is no need of high or low temperature, no additional radical matrix, no microwave, laser radiation or the necessity of expensive hardware, etc.

One prerequisite for the structural investigation of biological entities, e.g. peptides via PHIP is the implementation of unsaturated moieties, which can be hydrogenated by *para*-H_2_^15^.

To chemically functionalize natural proteins, the binding method and also the label itself must satisfy a number of requirements. In addition to biocompatibility, they must be stable and biologically safe. This implies that the label must have a high selectivity and reactivity towards a certain binding site, while preserving the tertiary structure^16^.

One approach is the use of tags (epitopes) to functionalize proteins^17^. Unfortunately, these labels are rather large, which can influence the molecule’s structural features. Furthermore, these tags bind specifically to a particular amino acid sequence. That’s why the target system must be known precisely in advance^17,18^. Besides this enzymatic approach, non-canonical amino acids that contain different functional groups (ethynyl-^19^, azido-^20^, halogen-^21^), can be incorporated into the peptide’s sequence and further utilized^12,15,22–33^. However, this process is exceptionally labor-intensive and proceeds with only low yields^34^.

**Scheme 1:**
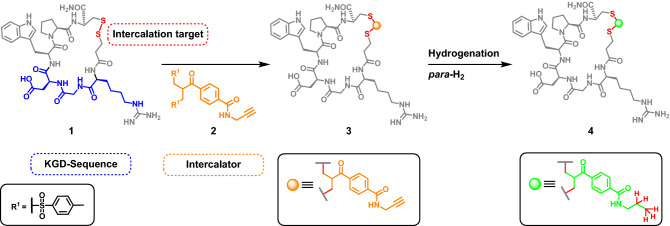
Illustration of the Intercalation-hydrogenation strategy to boost signal intensity in NMR experiments using the ethynyl-containing label **2**. Additionally, the biochemical relevant “K”GD-amino acid sequence ("Lys"-Gly-Asp) is highlighted. [We followed the usual nomenclature of the binding motif although it should read homo-R-G-D instead of K-G-D]^48,49^.

To overcome these issues, the post modification of peptides with PHIP-relevant fragments by small and selective labels is the most promising approach. Among the numerous possibilities that are already described in literature^35–39^, we decided to adopt the bifunctional cross-link strategy based on acylated allylic sulfones, which was first described by Liberatore and Brocchini^40,41^. They selectively modified disulfide bridges that are frequently present in therapeutically relevant proteins as binding sites, e.g. antibodies^41^ or somatostatin^16^. Those disulfide bonds are reduced and expanded by three carbon atoms, which can carry additional functional elements. The decisive advantage of this method is that the integrity of the protein structure isn’t compromised^42^. Weil et al. took advantage of this concept and presented a toolbox of allylsulfone intercalators presenting various functional groups such as boronic acid, azide or rhodamine as imaging probe^43^. Moreover, they prepared the ethynyl-containing intercalator **2**, which we utilized for our PHIP experiments (Scheme 1).

As a model system we chose the cyclic heptapeptide eptifibatide (Integrilin, **1**), which is derived from a disintegrin protein^44^ found in the venom of rattlesnake (s*istrurus miliarius barbouri)*, which is used as an antiplatelet aggregation inhibitor. The high potential of this drug is based on its cyclic structure and the "K"GD amino acid sequence (Lys-Gly-Asp)^45–47^.

Herein, we demonstrate by high performance liquid chromatography (HPLC) and electrospray ionization mass spectrometry (ESI–MS) that the intercalator **2** can be incorporated into the disulfide bridge of the peptide. Recently it has been shown that eptifibatide can be efficiently spin-labeled for hyperpolarization applications employing Dynamic Nuclear Polarization^50^. In the present paper we demonstrate that it is also feasible to employ derivatives of eptifibatide as efficient hyperpolarization sources in PHIP-NMR spectroscopy. In particular, we point out the high value of the label for PHIP-NMR spectroscopy by hydrogenation of the ethynyl fragment (Scheme 1).

## Methods

All synthetic procedures, reagents, methods as well as spectroscopic parameters and spectra of the PHIP-measurements are reported in the Supporting Information.

## Results and discussion

### Synthesis and intercalation of label 2

**Scheme 2:**
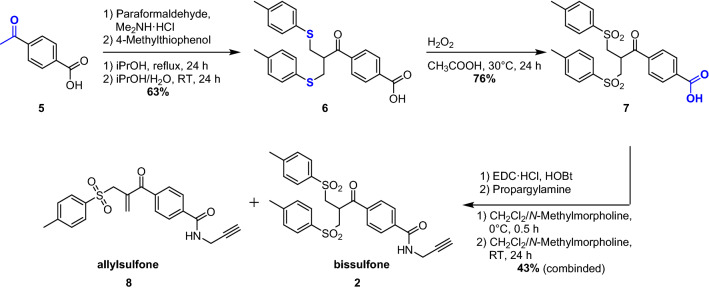
Synthesis of the intercalator mixture **2** and **8**.

**Figure 1:**
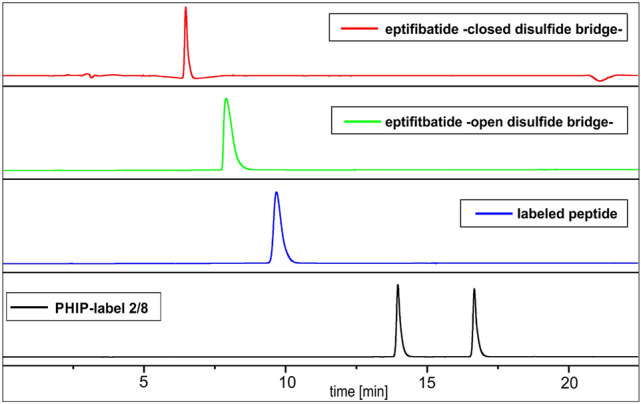
HPLC traces of the labeling process shown in Scheme 3 (solvent gradient of ACN/H_2_O: 20/80 to 80/20, λ = 214 nm).

According to Scheme 2, we started our research by synthesizing the bissulfone label **2** following a published procedure to a similar compound^51^ with slight modifications. In the first step *p*-acetyl benzoic acid **5** was subjected to a double Mannich-type reaction. The crude reaction product was directly used in the following substitution reaction with *p*-thiocresol as a nucleophile yielding the bisulfide **6** which, after recrystallization was isolated in 63% yield. After oxidation of the sulfides by peracetic acid the corresponding bissulfone **7** was obtained in 76% yield. The implementation of the PHIP-relevant ethynyl-fragment was achieved by EDC-mediated (1-Ethyl-3-(3-dimethylamino-propyl)carbodiimide) amide-coupling with propargylamine. After purification by column chromatography the intercalator was separated from all hazardous impurities as observed by analytical HPLC and HPLC–MS (Fig. 1 and Supplementary Fig. 7) and was obtained as a mixture of bissulfone **2** and allylsulfone **8** with a yield of 43% and an overall yield of the 3-step synthesis of 21%. Considering the fact that the bissulfone **2** will be converted into the allylsulfone **8** in the intercalation reaction anyway, no further purification is needed. (For more information see Ref.^43^ and Supplementary Information).

**Scheme 3:**
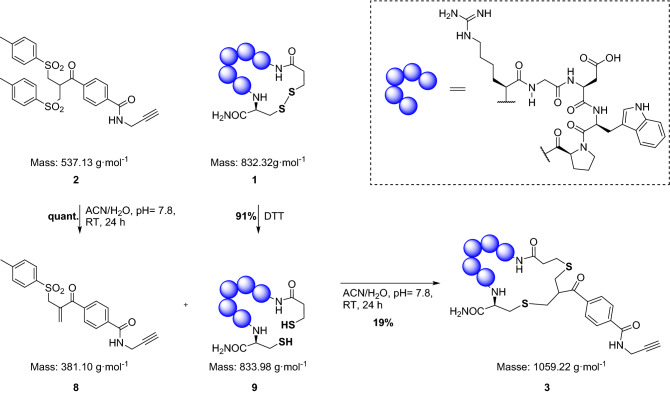
Illustration of the intercalation process of the label **2/8** into the in advance reduced disulfide bond of eptifibatide (DTT: Dithiothreitol).

With the label mixture (**2**/**8**) in hand, the intercalation reaction proceeded in a two-step sequence. First, the mixture of **2** and **8** is converted completely to the reactive species **8** by dissolving the mixture in a slightly alkaline aqueous solution (pH = 7.8). At this point the reduced eptifibatide (after purification) was added and the resulting mixture stirred at room temperature until full consumption of the starting materials (Scheme 3).

The insertion process of the unsaturated fragment into the disulfide bridge can be followed by HPLC (Fig. 1). The DTT-mediated (Dithiothreitol) reduction of eptifibatide results in a prolonged retention time of the resulting dithiol **9** compared to eptifibatide **1** with a closed disulfide bridge. Similarly, the insertion of the label **8** causes a delayed elution time for the modified peptide **3** relative to the dithiol **9**. The HPLC-trace of the intercalator mixture **2** and **8** is shown to verify its complete consumption.

**Figure 2:**
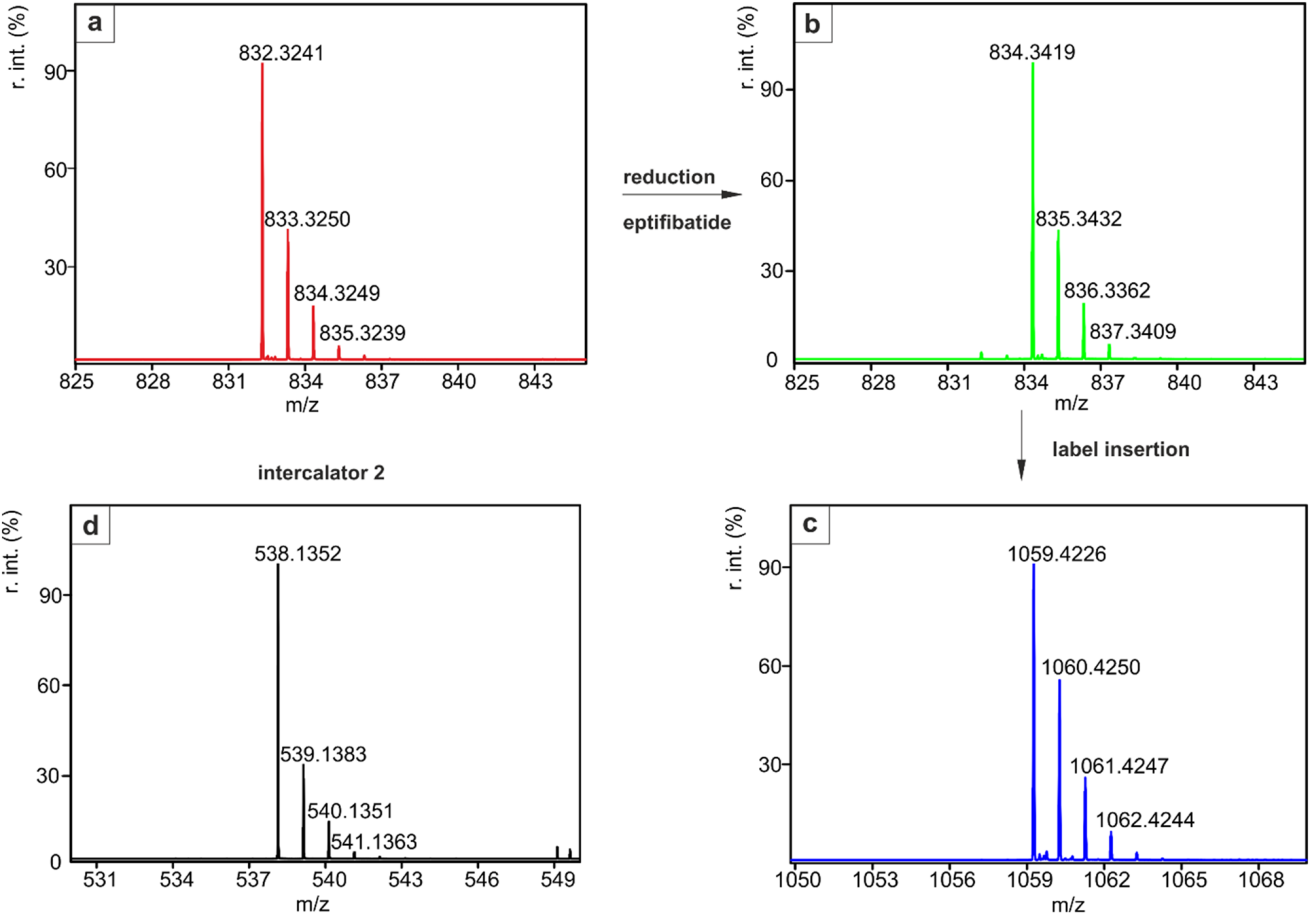
Relevant sections of the ESI–MS spectra recorded during the intercalation process as described in Scheme 3. [(**a**) Eptifibatide, (**b**) Reduced eptifibatide, (**c**) Eptifibatide after label insertion, (**d**) intercalator **2**/**8**].

In parallel to the HPLC traces, the successful incorporation of the label can also be confirmed by ESI–MS. Analogous to the chromatograms shown above, the molecular ion peaks are shown, which indicate the increase in mass of the peptide due to the binding of the label. The measured mass spectra agree with the theoretically determined values (Fig. 2 and Supplementary Information).

### Enhancement factor determination

**Scheme 4:**
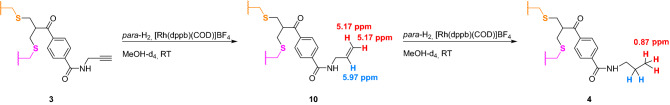
Hydrogenation of the triple bond containing PHIP-label inserted in the disulfide bridge of eptifibatide **3** with *para*-H_2_.

To show the suitability of the label **2** for PHIP-NMR spectroscopy, the triple bond is hydrogenated with *para*-H_2_ using [Rh(dppb)COD)]BF_4_ as catalyst (Scheme 4).

**Figure 3:**
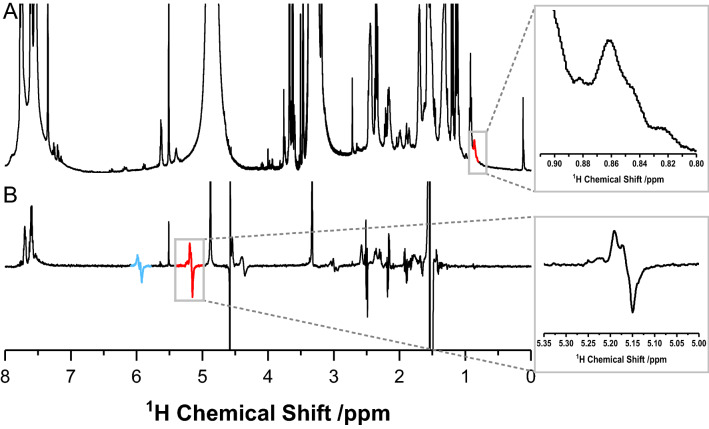
Comparison of the ^1^H-NMR spectra of (**A**) the thermally relaxed spectrum after full hydrogenation (4096 scans) and (**B**) the PHIP spectrum (single scan) after addition of *para*-H_2_ (enrichment of > 95%) (500 MHz, MeOH-d4, 298 K). The PHIP spectrum reveals hyperpolarized lines of the catalyst system (see Supplementary Fig. 10) and the strongly PHIP enhanced signal of the labeled eptifibatide, which is visible in the single scan spectrum with a good signal-to-noise ratio. The obtained enhancement factor, estimated from the insets for the signal at 5.17 ppm is above 1000 (see Supplementary Information for calculation).

For the determination of the enhancement factor several ^1^H-NMR spectra are acquired. In addition to the spectrum after complete hydrogenation (in thermal equilibrium structure **4**), a spectrum showing the short-lived first hydrogenation step to the parahydrogen added *N*-allyl amide **10** was recorded (Fig. 3B). Based on this hydrogenation the PHIP activity of the ethynyl labeled eptifibatide **3** was proven and an enhancement factor determined was estimated (see Supplementary Information for calculation).

Figure 3 shows the spectra of **3** in deuterated methanol under enhancement conditions (during hydrogenation with *para*-H_2_; Fig. 3B) and in thermal equilibrium after complete hydrogenation (Fig. 3A). The PHIP signals of the vinyl group are located at 5.95 and 5.17 ppm (terminal protons). The PHIP-signals with the low chemical shift and the complex multiplicity in the range from 4.38 to 1.54 ppm can be assigned to the 3 mg of the catalytically active complex Rh(dppb)(COD)BF_4_ contained in the sample (see Supplementary Information), the “pseudo” PHIP signal at 4.58 ppm is due to the PNL-Effect (partial negative line shape). This arises when molecular hydrogen enriched with *para*-H_2_ is solubilized in e.g., methanol interacting with an organometallic hydrogenation catalyst^52^.

The PHIP spectrum (Fig. 3B) is measured in a single scan. For the thermally relaxed spectrum (Fig. 3A), 4096 scans were required to observe a visible and evaluable signal of the hydrogenated label (the terminal methyl group of the fully hydrogenated product). The required high number of scans in the thermally relaxed spectrum is due to the low concentration of 0.2 mM of **3** in MeOH-d_4_.

Owing to the transient nature of the vinyl-intermediate, which is not visible in the thermally relaxed spectrum, the enhancement factor can be estimated only indirectly by comparing the integral ratios of the PHIP-spectrum (vinyl signal at 5.17 ppm) and the thermally relaxed spectrum (alkyl signal at 0.86 ppm), marked as insets in Fig. 3 and assigned in Scheme 4. Comparing these integrals, a factor of 1000 can be estimated as lower limit for the enhancement factor (details of the calculation can be found in Supplementary Information).

These results clearly prove that the insertion of label **2** was successful leading to a very efficient PHIP labelling. It has been demonstrated that PHIP-active, site selective labeled biomolecules can be synthesized using the approach described above. This PHIP labeling creates a very strong signal enhancement of the protons of the allyl-group. Such a signal enhancement can be employed for example for selective binding studies of the eptifibatide or similar molecules to a target protein^15^. Moreover this opens up the way to ULTRAFAST 2D-NMR of such complexes^31^.

## Conclusion

A bis-sulfone-based label was synthesized and its suitability for the site-selective insertion into the disulfide bridge of a bioactive peptide was demonstrated. The ethynyl-modified intercalator was obtained as a two-component mixture of the bissulfone **2** and its elimination product **8**, where both components can participate in the labeling reaction because the active MICHAEL acceptor **8** is formed by elimination under the reaction conditions. As an example for the feasibility of the obtained unsaturated system as a substrate for PHIP labeling studies, the disulfide bridge of eptifibatide **1**, a synthetic cyclic heptapeptide derived from a disintegrin protein from the venom of the rattlesnake *Sistrurus miliarius barbourin*, was chosen. After label insertion, it was possible to hyperpolarize the target molecule **3** by a hydrogenation reaction with *para*-H_2_. A factor of 1000 was estimated as a lower limit for the signal enhancement of the hyperpolarized protons of the newly formed vinyl group in comparison with the final formed alkyl group. This result paves the way for future applications of ethynyl-substituted systems like **2**/**8** to be successfully used for future PHIP-applications, utilizing the site selective opening of disulfide bridges in biologically active compounds.

## Supplementary Information

Supplementary Information.

## Abbreviations

NMR: Nuclear magnetic resonance
SEOP: Spin exchange optical pumping
DNP: Dynamic nuclear polarization
PHIP: *Para*Hydrogen induced polarization
SABRE: Signal amplification by reversible exchange
SE: Signal enhancement
