# Supplementary material for: A disintegrin derivative as a case study for PHIP labeling of disulfide bridged biomolecules
Source: Sci Rep. 2022 Feb 11;12:2337. doi: 10.1038/s41598-022-06327-z (PMC8837631; doi:10.1038/s41598-022-06327-z)
Supplement: Supplementary file 1 — Supplementary Information. [file 41598_2022_6327_MOESM1_ESM.pdf]

A disintegrin derivative as a case study for PHIP labeling of disulfide bridged biomolecules

Max Fleckenstein[a+], Kevin Herr[b+], Franziska Theiß[b], Stephan Knecht[b], Laura Wienands[b], Martin Brodrecht[b], Michael Reggelin[a] and Gerd Buntkowsky[b]**

Experimental Methods

a. General

Chemicals

All materials were purchased from commercial suppliers (Fisher Scientific, Acros Organics, Merck, ABCR, Alfa Aesar) and used without further purification unless otherwise described. Eptifibatide (CAS: 188627-80-7) was purchased from Ambeed, Inc.. Solvents were purchased HPLC grade and HOBt (1-Hydroxybenzotriazole) was dried by azeotropic codistillation with toluene. Deuterated solvents for NMR-spectroscopy were purchased from Sigma-Aldrich.

Thin layer chromatography

Thin layer chromatography was performed using silica gel (SilG/UV 252, plate thickness: 0.25 mm) by Macherey-Nagel GmbH & Co. KG. Visualization was achieved by UV fluorescence quenching or oxidizing with $KMnO_4$.

Nuclear Magnetic Resonance

^{1}H-NMR and ^{13}C-NMR-Spectra were recorded on Bruker ARX 300 or AC 300 operating at 300 MHz and 300 K, unless it's not otherwise specified. Chemical shifts (δ) are reported in ppm relative to TMS (δ = 0.00) or referenced to the residual solvent signal.[1] The fine structure of proton signals was specified as s for singlet, d for doublet, t for triplet, q for quartet or m for multiplet and b before the descriptor indicates broad signals.

PHIP-NMR

NMR experiments were performed on a Bruker Avance III NMR spectrometer at 11.7 T (500 MHz) in 528-TR-7 NMR tubes (5 mm [outer diameter]) from Rototec Spintec. For hydrogenation reactions, *para*-enriched hydrogen gas was bubbled through a 1/16 inch Teflon tubing (1.6 mm [outer diameter], 0.5 mm [inner diameter]) ending in a glass capillary (0.36 mm [outer diameter], 0.15 mm [inner diameter]). The end of the glass capillary is located in the detection region. For more information about the PHIP-setup and the measurement conditions, see ref[2].

Infrared spectroscopy

All solids were applied as a thin film to the measurement window of the spectrometer (Spectrum II FT-IR-Spectrometer -PerkinElmer-) and the IR spectra were accumulated subsequently.

Mass spectrometry

EI-MS-spectra were recorded on a double focussing Finnigan MAT 95 spectrometer. The APCI and ESI measurements were acquired on an Impact II spectrometer (Bruker). The detected ions are denoted in *u* relative to the strongest signal.

Reverse-Phase High-Performance Liquid Chromatography (RP-HPLC)

Reversed phase high-performance liquid chromatography (RP-HPLC) for analytical analysis was conducted using a Waters HPLC Setup consisting of a Waters Alliance e2695 equipped with a Waters 2998 PDA detector. The detection wave lengths were 214, 254 and 301 nm. The eluent system for the HPLC system comprised eluent A (0.1% aq. TFA) and eluent B (99.9% acetonitrile containing 0.1% TFA). Analytical HPLC runs were conducted at a flow rate of 1 ml/min with an eluent gradient from 20 % to 80 % of eluent A over 20 min and flush step for 10

min with 95% of eluent A for peptides and the PHIP label. For all analyses a Nucleosil 100–5C18 column from Macherey-Nagel (5 µm, 100 Å) was used. Preparative purification was performed on a Knauer Multokrom RP18 column 20×250 mm (5 µm, 100 Å) employing a flow rate of 9 mL/min and an acetonitril gradient from 20% to 80% within 60 min

Elemental analysis

Elemental analysis was performed on a Vario EL III (Elementar).

Melting point measurement

Melting points were measured with a melting point apparatus from HWS-Laboratoriumstechnik.

b. Label synthesis

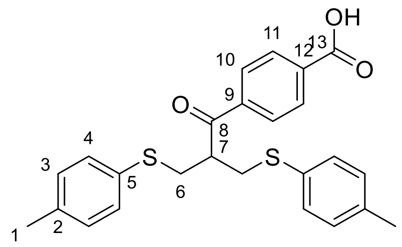

6

Bisthioether (6): According to the literature[3] 5.66 g (34.48mmol, 1.00 Eq.) 4-acetyl benzoicacid, 5.01 g (167.0 mmol, 4.84 Eq.) paraformaldehyde and 13.62 g (167.0 mmol, 4.84 Eq.) dimethylamine hydrochloride were diluted in iso-propanol and heated to reflux for 24 h. Upon cooling a precipitate appears, which was redissolved by adding H_2O. After adding 8.56 g (68.95 mmol, 2.00 Eq.) 4-methyl benzenethiol an Ar-atmosphere was applied. The reaction mixture was heated to reflux again for 24 h and the precipitated solid filtered off after cooling to room temperature. The resulting solid was rinsed with water, dried and recrystallized in methanol to obtain the product as a colorless solid. Yield: 9.47 g (63%); ^{1}H-NMR (300 MHz, $CDCl_3$, 301.2 K): δ (ppm) = 11.11 (bs, 1-COOH), 8.06 (d, ^3J = 8.3 Hz, 2-H_{10}), 7.63 (d, ^3J = 8.3 Hz, 2-H_{11}), 7.15 (d, ^3J = 7.98 Hz, 4-H_4), 7.06 (d, 7.98 Hz, 4-H_3), 3.83 (p, 1-H_7), 3.22 (m, 4-H_6), 2.36 (s, 6-H_1); ^{13}C-NMR (75 MHz, $CDCl_3$, 301.2 K): δ (ppm) = 200.0 (8-C), 170.9 (13-C), 140.5 (2-C), 137.2(9-C), 132.8 (12-C), 131.5 (3-C), 131.0 (5-C), 130.2 (11-C), 129.8 (4- C), 128.3 (10-C), 45.9 (7-C), 36.3 (6-C), 21.1 (1-C); EA ($C_{25}H_{24}O_3S_2$): calculated: C: 68.78 H: 5.54 N: 0.00, measured: C: 68.95 H: 5.47 N: 0.00; M_p.: 137.5-140.0°C.

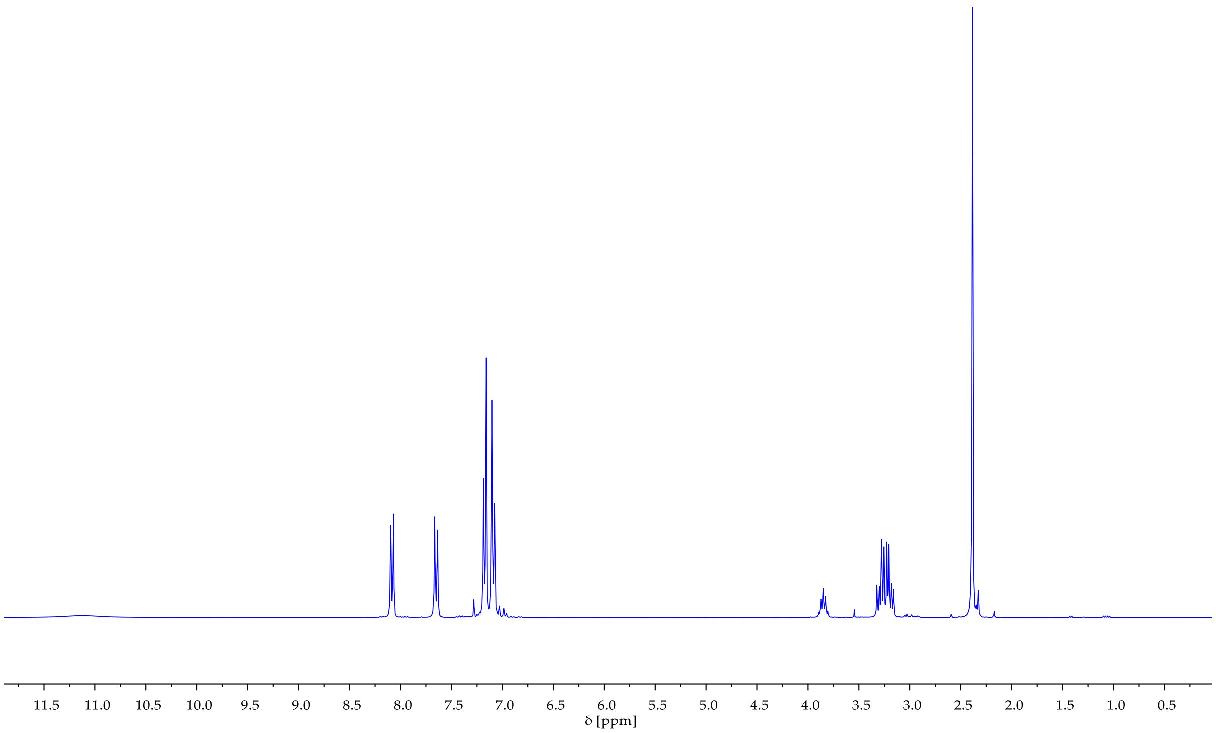

Figure 1: ^{1}H-NMR-Spectrum of bisthioether **6**in $CDCl_3$ at 301.2 K and 300 MHz.

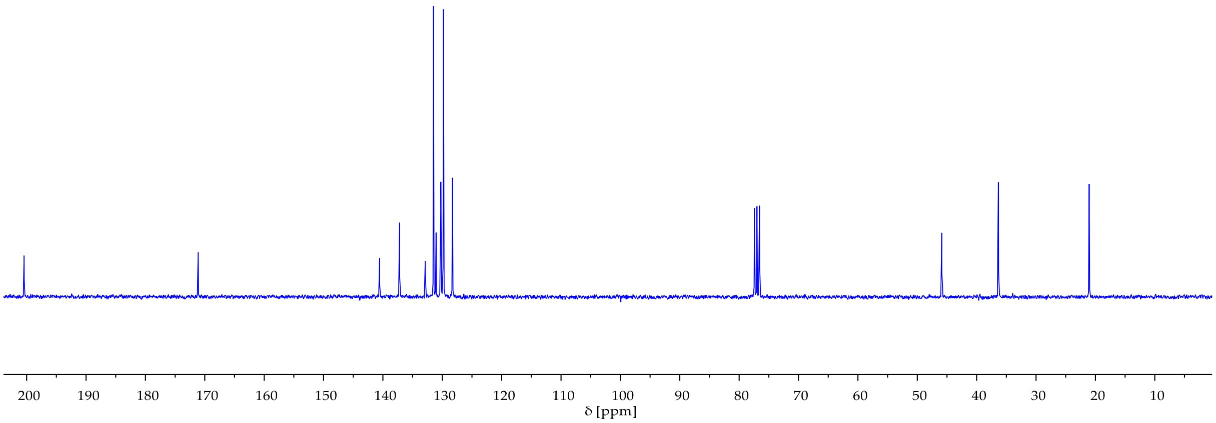

Figure 2: ^{13}C-NMR-Spectrum of bisthioether **6** in $CDCl_3$ at 301.2 K and 75 MHz.

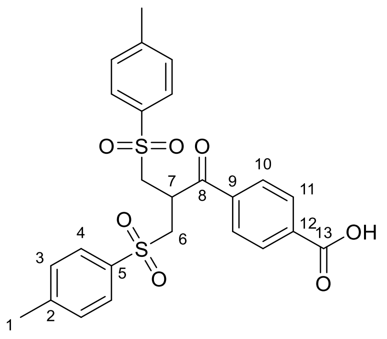

7

4-(3-tosyl-2-(tosylmethyl)propanoyl)benzoic acid (7): 3.00 g (6.87mmol, 1.00 Eq.) of the bisthioether **6** was dissolved in 100 mL acetic acid and heated to 30°C.[3] After adding 4.56 mL (44.67 mmol, 6.50 Eq.) H_2O_2 (30-35%) the resulting mixture was stirred overnight. Diethylether (50 mL) and water (100 mL) were added, the phases were separated, and the aqueous phase was extracted another two times with ether. The combined organic extracts were washed with saturated solution of $NaHSO_3$ and brine. The organic phase is dried over Na_2SO_4, filtered and the solvent removed under reduced pressure. The crude product was recrystallized in water. A colorless solid was obtained. **Yield**: 2.60 g (76%), **^{1}H-NMR** (300 MHz, DMSO-d_6, 303K): δ (ppm) l= 13.35 (bs, 1-COOH), 7.95 (d, 2-H_{10}, ^3J = 8.63 Hz), 7.60-7.50(m, 6-$H_{4, 11}$), 7.43(d, 2-H_3, ^3J = 8.39 Hz), 3.99 (p, 1-H_7), 3.89-3.65 (m, 4-H_6), 2.45 (s, 6-H_1); **^{13}C-NMR** (75 MHz, $CDCl_3$, 301.2 K): δ (ppm) 195.5 (8-C), 166.3 (13-C), 145.1 (2-C), 137.8 (9-C), 135.9 (5-C), 130.1 (3-C), 129.4 (10-C), 128.2 (11-C), 127.8 (4-C), 54.9 (6-C), 35.2 (7-C), 20.9 (1-C); EI-MS: 344 (5, $[M-C_7H_7O_2S]^+$), 149 (100, $[M-C_{17}H_{19}O_4S_2]^+$), 91 (95, $[M-C_{18}H_{17}O_7S_2]^+$); **EA** ($C_{25}H_{24}O_3S_2$): calculated: C: 59.98 H: 4.83 N: 0.00, measured: C: 59.96, H: 4.89, N: 0.00; **M$_p$.**: 169.0-172.5°C.

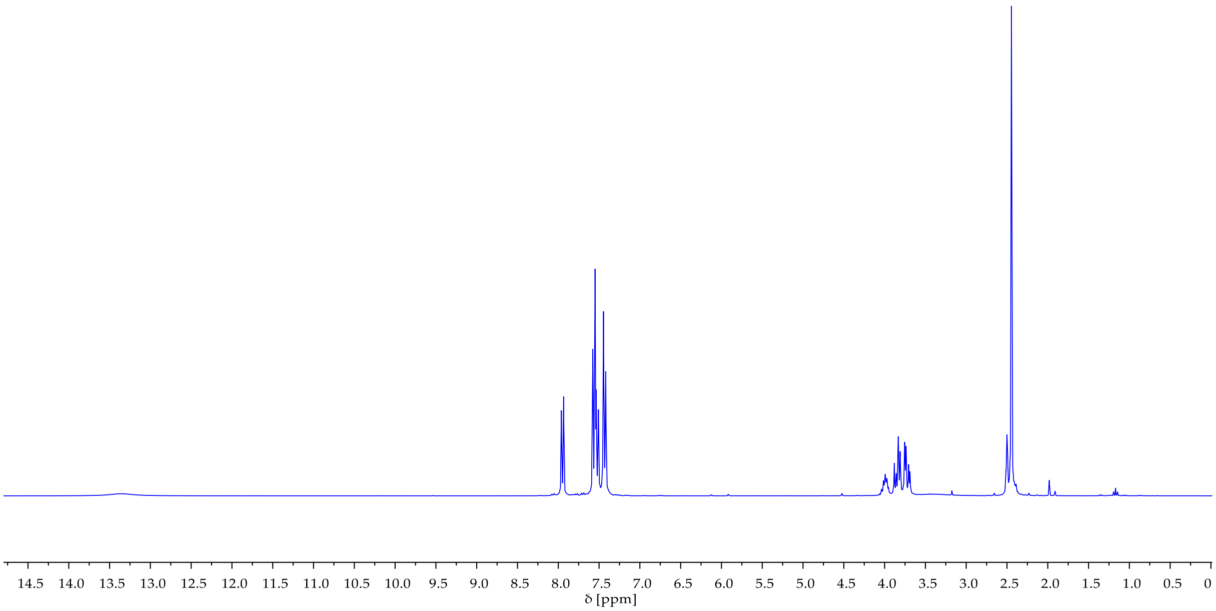

Figure 3: ^{1}H-NMR-Spectrum of bissulfone **7** in DMSO-d_6 at 303 K and 300 MHz.

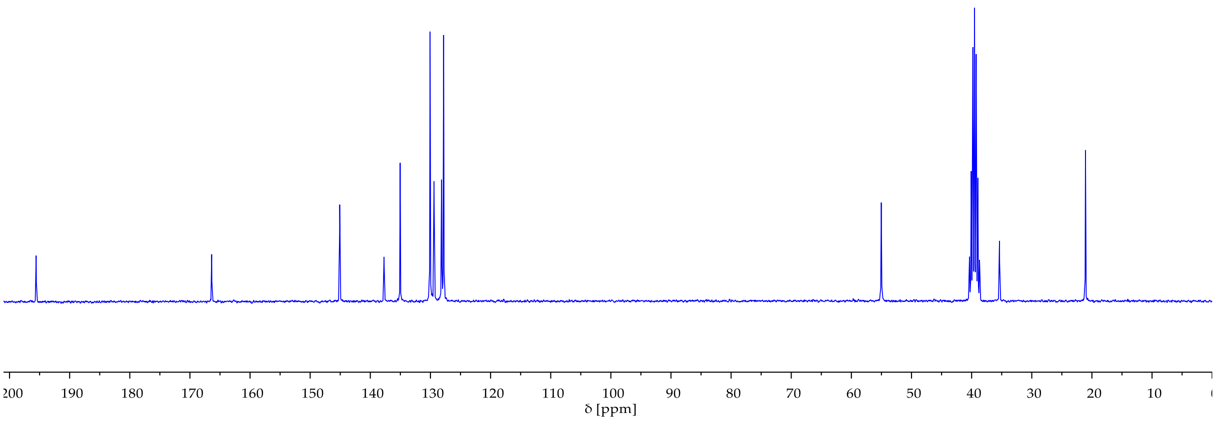

Figure 4: ^{13}C-NMR-Spectrum of bissulfone **7** in DMSO-d_6 at 303 K and 75 MHz.

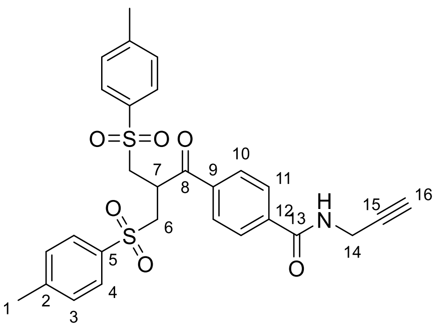

2

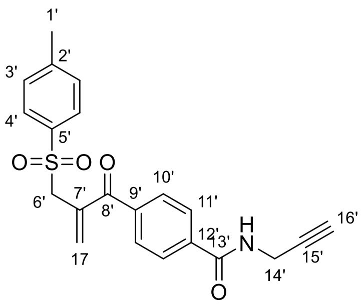

8

Ethynyl-containing Label (2/8): In a 100 mL round bottom flask 500 mg (0.99 mmol, 1.00 Eq.) of bissulfone **7** and 0.22 mL (1.99 mmol, 2.00 Eq.) *N*-methylmorpholine were dissolved in 25 mL methylene chloride and cooled to 0°C. After adding 148.5 mg (1.099 mmol, 1.10 Eq.) HOBt and 229.8 mg (1.20 mmol, 1.20 Eq.) EDC·HCl the reaction was stirred at 0°C until TLC shows complete conversion. 55 mg (0.99 mmol, 1.00 Eq.) propagylamine were added and stirred overnight, while warming up to room temperature. To isolate the product water (50 mL) was added and the phases were separated. The aqueous phase was extracted another two times with methylene chloride and the combined organic phases were washed with water and brine. Na_2SO_4 was added to the organic phase, filtered and solvent removed under reduced pressure. The crude product was purified by flash chromatography on silica (EA:PE/1:1). A colorless foam was obtained, which was a mixture of allylsulfone **8** and bissulfone **2**. **Yield**: 230 mg (43%); **R$_f$** (EA:PE/1:1): 0.20 (allylsulfone), 0.30 (bissulfone); **^{1}H-NMR** (300 MHz, $CDCl_3$, 303K): δ (ppm) = 7.84 (d, ^3J = 8.38 Hz, 1-H_{10}), 7.78 (d, ^3J = 8.13 Hz, 1-$H_{10'}$), 7.77 (d, ^3J = 8.38 Hz, 1-H_{11}), 7.72 (d, ^3J = 8.13 Hz, 1-$H_{11'}$), 7.69 (m, 6-$H_{4,4'}$), 7.35 (d, 6-$H_{3,3'}$, ^3J = 8.31 Hz), 6.51 (t, 1-NH), 6.47 (t, 1-NH), 6.26 (s, 1-H_{17}), 5.97 (s, 1-H_{17}), 4.34 (p, 1-H_7), 4.33 (s, 2-$H_{6'}$), 4.28-4.24 (m, 4-$H_{14,14'}$), 3.66-3.44 (m, 4-H_6), 2.47 (s, 6-H_1), 2.41 (s, 3-$H_{1'}$), 2.33-2.28 (m, 2-$H_{16,16'}$); **^{13}C-NMR** (75 MHz, $CDCl_3$, 303K): δ (ppm) = 195.3 (8-C), 194.2 (8'-C),

166.2 (13-C), 165.5(13'-C), 145.7 (5-C), 145.3 (5'-C), 139.2 (12-C), 137.3 (12'-C), 136.8 (7'-C), 136.0 (9'-C), 135.9 (9-C), 135.4 (Σ 2, 2'-C), 134.5 (17-C), 130.3 (3-C), 130.1 (3'-C), 129.9 (11'-C), 128.9 (11-C), 128.4 (Σ 4-C, 4'-C), 127.7 (10'-C), 127.1 (10-C), 79.2(15-C), 79.1 (15'-C), 72.4 (16-C), 72.3 (16'-C), 57.8 (6'-C), 55.8 (6-C), 35.7 (7-C), 30.1 (Σ 14-C, 14'-C), 21.9 (1-C), 21.8 (1'-C); **EI-MS**: 537 (5, $[M]^+$), 381 (70, $[M-C_7H_7O_2S]^+$), 186 (100, $[M-C_{17}H_{21}O_4S_2]^+$), 91 (95, $[M-C_{21}H_{20}NO_6S_2]^+$); **EI-HRMS** ($C_{28}H_{27}NO_6S_2$): measured: 537.1273 $[M]^+$ (bissulfone), calculated: 537.1274 $[M]^+$ (bissulfone); **IR** : ṽ = 1651, 1529, 1288, 1084 cm^{-1}; **HPLC:** t_R = 13.9 min, t_R = 16.7 min.

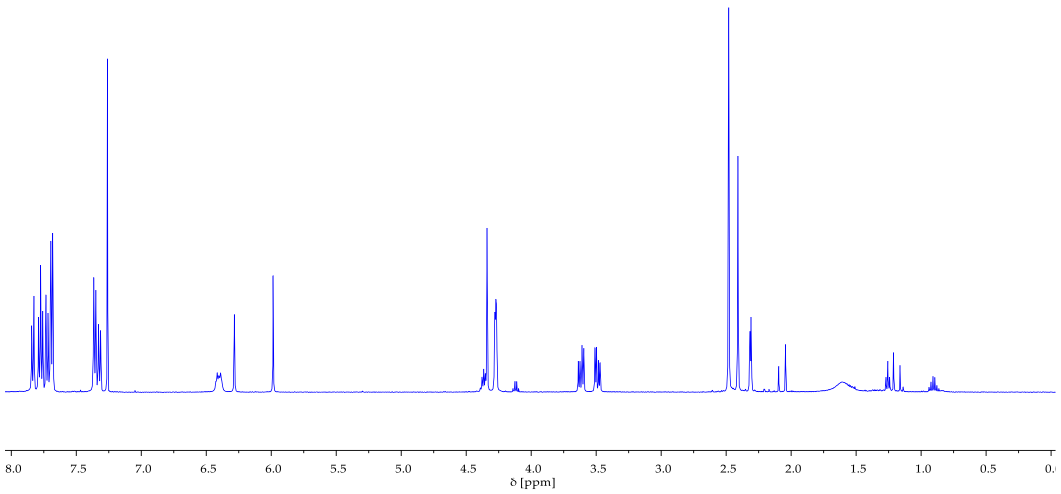

Figure 5: ^{1}H-NMR-Spectrum of Label **2/8** in $CDCl_3$ at 303 K and 300 MHz.

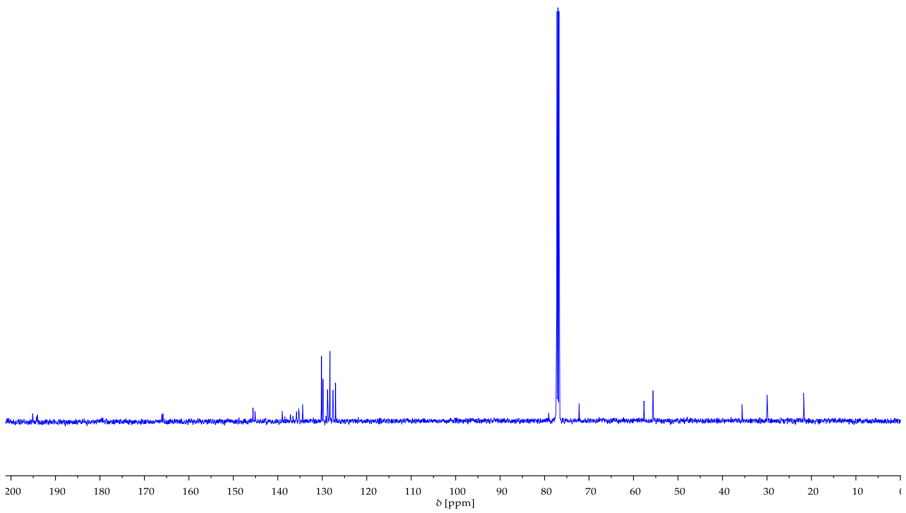

Figure 6: ^{13}C-NMR-Spectrum of Label **2/8** in $CDCl_3$ at 303 K and 75 MHz.

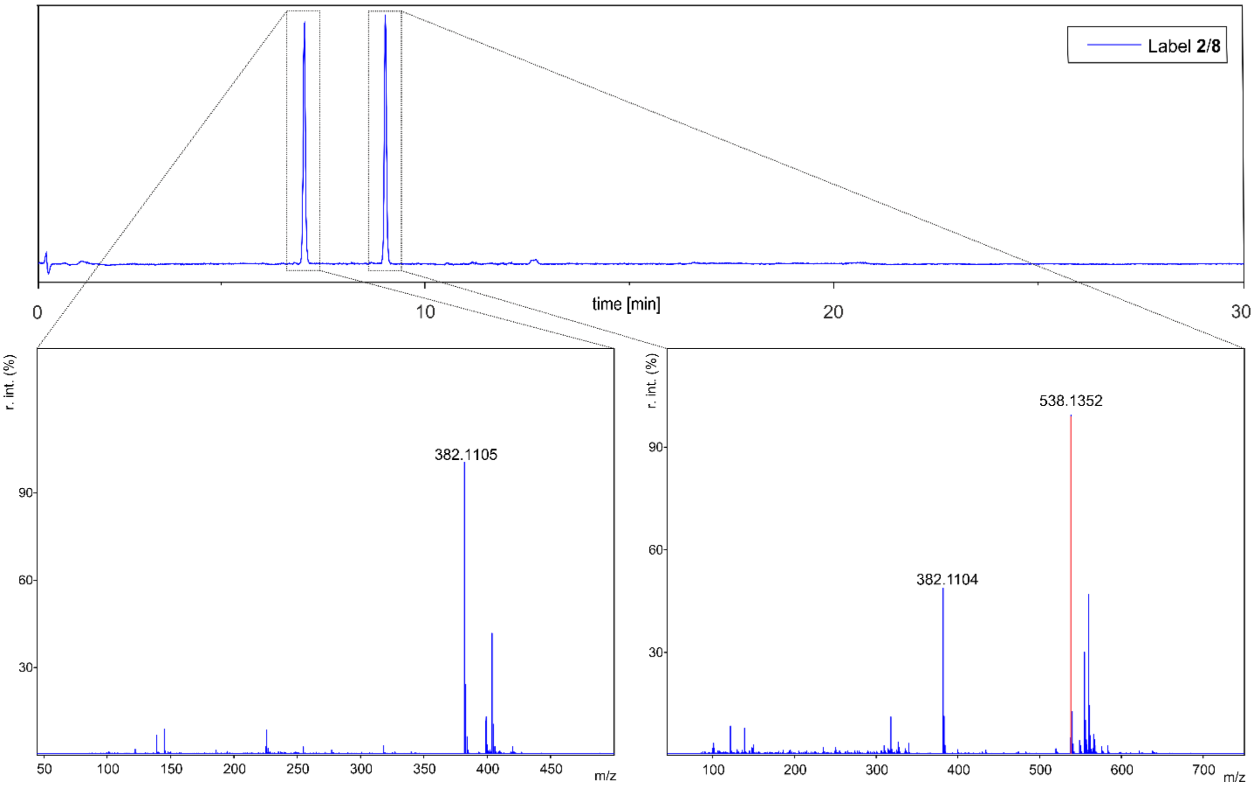

Figure 7: HPLC-MS-Data for Label **2/8**.

c. Peptide modification

Reduction of eptifibatide: Dithiol (9): 28 mg (33 µmol, 1.00 Eq.) of eptifibatide **1** is dissolved in 5.0 mL acetonitrile and 5 mL anal. pur. water. Then 20.6 mg (132 µmol, 4.00 Eq.) of dithiothreitol were added and the mixture was stirred for 21 h at room temperature. To complete the reaction another 10.2 mg (66 µmol, 2.00 Eq.) of dithiothreitol were added and the mixture was stirred for 2 more hours at room temperature. Then the solvent was removed under reduced pressure and the crude product was purified by HPLC. **Yield**: 25.6 mg; **HPLC:** t_R = 7.9 min; **EI-MS**: $[M]^+$ = 834.34 m/z (simulated 834.34 m/z)

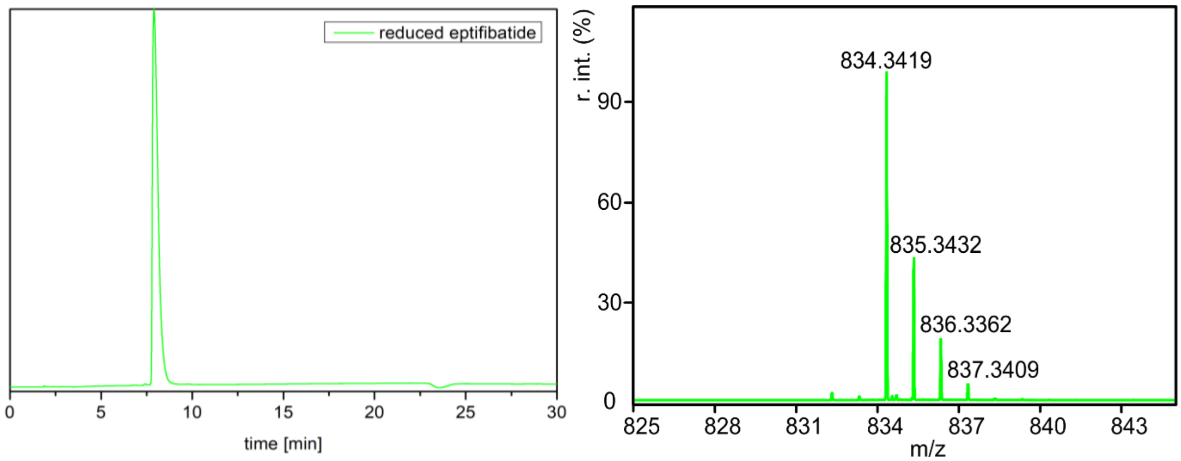

Figure 8: Mass spectrum and HPLC-Chromatogram of reduced eptifibatide (dithiol **9**; detected at 214nm).

Labeling of reduced eptifibatide 9 with bissulfone 2/8: According to literature[4] 11.6 mg (22 µmol, 2.00 Eq.) of the label **2/8** is dissolved in 3.9 mL acetonitrile and 6 mL phosphate buffer (pH = 7.8, c = 50 mM) and stirred for 24 h at room temperature. After addition of 9.00 mg (11 µmol, 1.00 Eq.) of reduced eptifibatide **9** dissolved in 1.8 mL acetonitrile and

2.7 mL phosphate buffer the mixture was stirred for another 24 h. Then the solvent was removed under reduced pressure and the crude product purified by HPLC **Yield**: 1.5 mg (19 %); **HPLC:** t_R = 9.4 min; **EI-MS**: $[M]^+$ = 1059.42 m/z (simulated 1059.42 m/z)

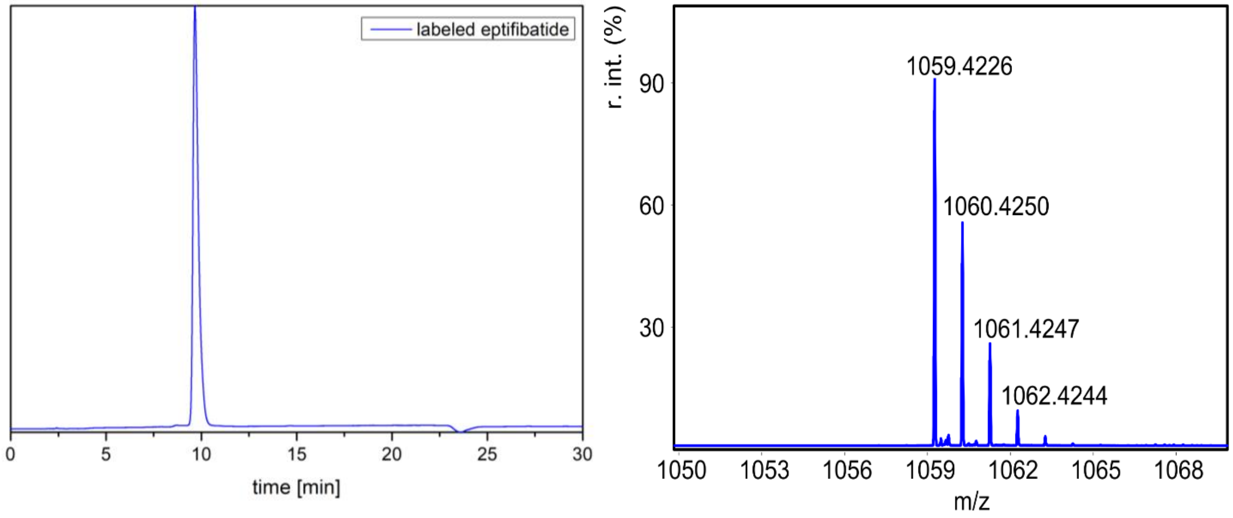

Figure 9: Mass spectrum and HPLC-Chromatogram of labeled eptifibatide **3** (detected at 214 nm).

d. PHIP-NMR experiments

For the PHIP experiments the setup described by Kiryutin et al[2] was used. Therefore 0.1 mg labeled eptifibatide and 3.0 mg of rhodium catalyst ([1,4-Bis(diphenylphosphino)butane](1,5-cyclooctadiene)rhodium(I) tetrafluoroborate; [Rh(dppb)(COD)]BF_4) were dissolved in 500 µL methanol. For 10 seconds *para*-enriched hydrogen (> 95%) was bubbled through the sample with a pressure of 5 bar, measured at the gas outlet of the NMR tube, at 25°C. At the end of the hydrogenation reaction, Helium is conducted into the NMR-tube. After stopping the hydrogen gas flow and changing to a static Helium pressure, a ^{1}H-NMR spectra (1 scan) was recorded. In addition to this spectrum, called PHIP-spectrum, another spectrum is recorded after the hyperpolarization has subsided (thermally relaxed spectrum). For comparison of the signals a further couple of spectra was recorded. The number of scans were adjusted individually.

For the sake of completeness, a spectrum of the PHIP-mixture is recorded before hydrogenation. This contains the catalyst and the still untouched labeled eptifibatide (**3**, in MeOD-d_4) (see figure 10 °C).

The single Rhodium catalyst (in MeOD-d_4) (see Figure 10 D) allows the assignment to the catalyst specific signals, which also occur during hyperpolarization. The catalyst signals display a low chemical shift (4.38 ppm to 1.54 ppm) and the complex multiplicity.

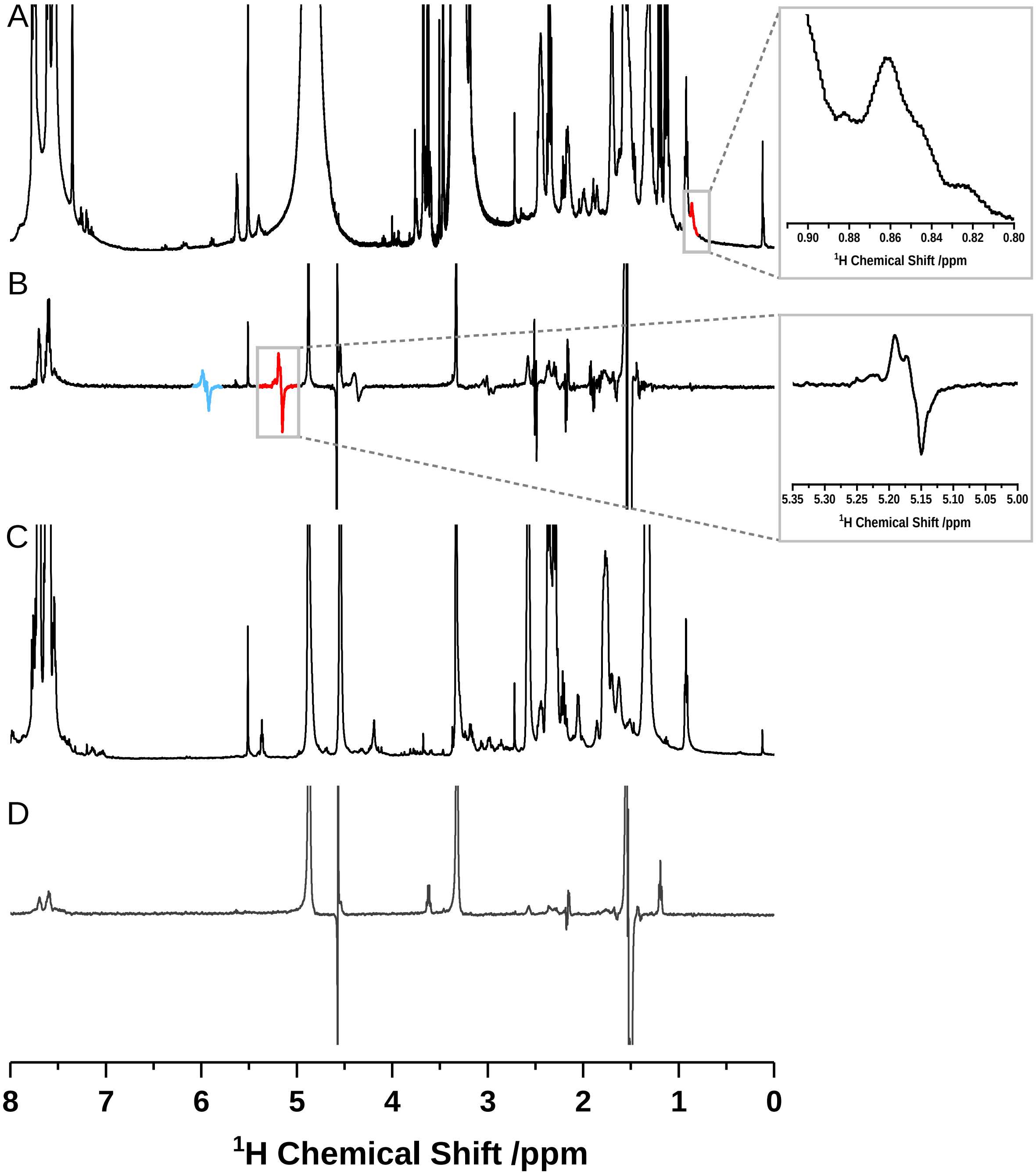

Figure 10: Comparison of the ^{1}H-NMR spectra of (A) the thermally relaxed spectrum after full hydrogenation and (B) after addition of *para*-H_2 for 10 sec. (C) the spectrum of labeled eptifibatide **3** before hydrogenation and (D) the PHIP-spectrum with the dissolved catalyst but without the eptifibatide (500 MHz, MeOH-d_4, 298 K).

e. Calculation of the enhancement

To calculate the enhancement of the hydrogenation, the PHIP- and the thermally relaxed spectrum received a phase- and baseline correction in order to compensate for dead-time effects and other spectral artifacts.

A direct determination of the enhancement-factor by comparing the intensity of the hyperpolarized and relaxed allyl-moiety is not feasible, since the latter had already been further converted to alkyl (0.86 ppm) after the reaction termination. For this reason, we had to employ the signal of the alkyl-moiety as a measure of the relaxed spectral intensity. Because the alkyl signal (as seen in the enlargement) is positioned very close to another signal it is not clearly delineated and thus broadened, the determination of the relaxed signal is not unambiguous and the enhancement factor can be estimated only approximately by calculating the line-integrals employing MestReNova 11.0. Similarly, the line integral of the dispersion signal (5.17 ppm) of the Allyl group is calculated. The resulting integrals are adjusted with the number of protons, number of scans and the receiver gain. The quotient of the normalized intensities results in the amplification factor.

Employing this procedure an amplification of more than 1000 is estimated.

The comparison of the temporary allyl dispersion signal and the thermal alkyl is possible due to the reaction course.

REFERENCES

1. Fulmer, G. R.; Miller, A. J. M.; Sherden, N. H.; Gottlieb, H. E.; Nudelman, A.; Stoltz, B. M.; Bercaw, J. E.; Goldberg, K. I., NMR Chemical Shifts of Trace Impurities: Common Laboratory Solvents, Organics, and Gases in Deuterated Solvents Relevant to the Organometallic Chemist. *Organometallics* **2010,** *29* (9), 2176-2179.
2. Kiryutin, A. S.; Sauer, G.; Hadjiali, S.; Yurkovskaya, A. V.; Breitzke, H.; Buntkowsky, G., A highly versatile automatized setup for quantitative measurements of PHIP enhancements. *J Magn Reson* **2017,** *285*, 26-36.
3. McDowall, L.; Stenzel, M. H., Disulfide bridge based conjugation of peptides to RAFT polymers. *Polym. Chem.* **2014,** *5* (5), 1772-1781.
4. Wang, T.; Wu, Y.; Kuan, S. L.; Dumele, O.; Lamla, M.; Ng, D. Y. W.; Arzt, M.; Thomas, J.; Mueller, J. O.; Barner-Kowollik, C.; Weil, T., A Disulfide Intercalator Toolbox for the Site-Directed Modification of Polypeptides. *Chem. - Eur. J.* **2015,** *21* (1), 228-238.
